# Positive Orientation—a Common Base for Hedonistic and Eudemonistic Happiness?

**DOI:** 10.1007/s11482-017-9508-9

**Published:** 2017-02-03

**Authors:** Piotr Oleś, Tomasz Jankowski

**Affiliations:** Department of Personality Psychology, Institute of Psychology, John Paul II Catholic University of Lublin, Al. Raclawickie 14, 20-850 Lublin, Poland

**Keywords:** Happiness, Positive orientation, Meaning of life, Satisfaction with life, Optimism, Self-esteem

## Abstract

Positive orientation (PO) is proposed as a common base for hedonistic and eudemonistic senses of happiness. PO involves a tendency to formulate positive judgments concerning the self, one’s personal life, and the future. Previously, PO had been investigated in the context of the hedonistic approach to well-being. In this article, we tested a broader understanding of PO, which is conceptualized, here, as a latent factor underlying variables that exemplify hedonistic and eudemonistic view on happiness. Using two samples (*N* = 159 and *N* = 200), we tested three models of PO extended to include various measures of meaning of life. The extended models fitted the data well. Results suggest that PO can be a general factor that is the basis for integrating two aspects of well-being: searching for positivity and pleasure, as well as striving for meaning.

Philosophical notions of happiness refer to two influential traditions of thinking about people and the conditions required for a happy life. The first has been expressed, for example, in Epicurean philosophy, which introduces pleasure as the main source of happiness. Another has been propagated by Aristotelian philosophers, among others, and presents happiness as an essential feature of a fruitful life and as the result of engagement in valuable goals. While the former concept emphasizes pleasure, the latter emphasizes virtue (Ryan et al. 2008). In other words, people are able to experience well-being and gain happiness by striving for pleasure, by engaging in activities that follows their intrinsic motivation, or by striving for meaning, pursuing a purposeful life through the development of virtues (Peterson et al. 2005). Thus, three sources of happiness, in the widest sense, understood just as a “good life,” have been mentioned: pleasure, engagement, and meaning (Seligman 2002).

Both traditions of thinking about happiness have influenced psychology. Happiness has been defined in terms of pleasant life (e.g., Kahneman et al. 1999), as well as a meaningful life and engagement in life (e.g., Ryff 2014); however, various psychological theories emphasize either the former or latter understanding of well-being, but seldom both to an equal extent (Bandura 2001; Frankl 1984; Fredrikson and Losada 2005; Jacobsen 2007; Ryff 2014). The unbalanced perspective on well-being present in many theoretical approaches has led to the prevailing view that there are two distinct, and even opposing, perspectives on human wellness (Ryan et al. 2008). While in contemporary psychology a vast amount of knowledge has been gained about different aspects of wellness, there is also a need to understand some general form of happiness. In this article we suggest the existence of some unifying basis for happiness—a “positivity” factor—that influences both aspects of well-being: hedonic as well eudemonic. In other words we investigate whether there is a common basis for different aspects of happiness, namely a broad, latent factor, which underlies positive affect and pleasure, the search for meaning in life, and engagement in the fulfillment of personal goals.

The first attempts to identify a more general aspect of well-being have recently been made. After several decades of focusing on well-defined particular variables related to hedonia, such as satisfaction with life or optimism, a new trend has emerged, searching for a common basis for sets of variables representing some aspects of personal life and the self. This trend can be illustrated by core valuations (Judge et al. 2003), positive orientation (Caprara 2009), or quality of life (Land et al. 2012; Oleś 2014). Similar attempts have also been made with reference to eudaimonia. For example, Reker and Fry (2003) proposed a construct of existential meaning, which is derived from a hierarchical confirmatory model, and refers to a general, second order factor explaining common variance among six aspects of personal meaning measured by different methods. However, to our best knowledge, there has been made no research confirming hypotheses about a common basis for all aspects of well-being: pleasure, meaning of life, and engagement in life. The aim of this research is to propose the models that integrate all these perspectives.

At the start we refer to *positive orientation*, a concept recently introduced by Caprara (2009). Positive orientation refers to the observation that variables regarded as cognitive in their origin—self-esteem, satisfaction with life, and optimism—turned out to be interconnected and inherited to a substantial extent (Caprara et al. 2009). Thus, positive orientation is defined as a general latent variable underlying a common basis for self-esteem, satisfaction with life, and optimism. In other words, it is a general tendency to formulate positive judgments concerning the self, one’s personal life, and one’s personal future (Caprara 2009; Caprara et al. 2010). As self-esteem, optimism, and satisfaction with life are usually investigated within the “hedonic” approach, and they are main facets of hedonia, positive orientation can, thus, be understood as a construct explaining a general positive approach to self and life, which influences the level of various, more specific aspects of hedonic well-being. However, the question arises as to whether there is an even more general factor of positivity, which explains what both hedonic and eudaimonic aspects of happiness have in common. We think that Caprara’s original model of positive orientation can be extended to include both sense of meaning in life and life engagement.

There are several reasons to suggest that meaning of life and engagement in life can be further facets of positive orientation, together with the aforementioned triad. Results of several studies suggest that the two kinds of variables (hedonic vs eudaimonic) cannot be seen as opposing or unrelated, but, rather, they should be understood as positively bound together with each other. Meaning in life correlates with self-esteem, satisfaction with life, and optimism, to a moderate extent (e.g., Feder et al. 2008; Heisel and Flett 2004; Ho et al. 2010; Lyubomirsky et al. 2006; Pinquart and Frohlich 2009; Steger and Frazier 2005; Steger and Kashdan 2007; Vaughan and Kinnier 1996). Life engagement is also moderately correlated with optimism, satisfaction with life, and self-esteem (Scheier et al. 2006). Moreover, positive orientation, as well as meaning of life and life engagement, correlates with positive affect (Schlegel et al. 2009; Oleś et al. 2013; Scheier et al. 2006). Despite an obvious difference between happiness and meaning of life (Delle Fave et al. 2011; Schueller and Seligman 2010), according to a broad overview of the results (Heintzelman and King 2014), most people assess themselves as rather happy and, at the same time, regard their life as meaningful. Recently, several studies confirmed a close affinity between eudemonic and hedonic aspects of happiness (e.g., Deci and Ryan 2008; Vella-Brodric et al. 2009; Schueller and Seligman 2010). Such a pattern of results can be easily explained if we assume that optimism enables the person to engage in subjectively validated goals, self-esteem supports assertive strivings toward them, and satisfaction with life is a consequence of such activity. One might state that people are happy when they perceive their life as purposeful, significant, and comprehensible (regular, ordered, predictable, and saturated by reliable connections), as well as having a sense of their life being meaningful when they are happy (Heintzelman and King 2014).

On the other hand, moderate correlation between variables suggests that self-esteem, optimism, and satisfaction with life can also originate from a pleasant and comfortable life, and need not necessarily be related to an intensive and active life or to engagement in valuable goals. Moreover, a basic tendency to assess the self, life, and future in a positive way can be enhanced just by “positive illusions” (Taylor and Brown 1988). However, in order to avoid oversimplification, one should note that meaning of life usually implies reflection on life values more than assessment of self-esteem, optimism, or satisfaction with life. Thus, the possible inclusion of meaning of life as an aspect of positive orientation is not so obvious.

Arguments presented above allow for the treatment of hedonia and eudaimonia as different but related aspects of well-being. Significant and positive relationships between variables representing the hedonic and eudaimonic approaches to well-being suggest the existence of a basic factor explaining common variance shared by the aforementioned variables. Personal tendencies to formulate positive evaluations of life, future, and the self, as well as a sense of meaning and life engagement, can all be anchored in generally positive inclinations to assess crucial aspects of existence in similar ways.

It implies that the following research question is worth investigation: Do meaning of life and life engagement partly constitute positive orientation, together with the aforementioned triad? Our hypothesis is that all these variables are influenced by a common, latent factor of the second order—positive orientation. It seems likely to be the case, since they all represent core beliefs about the self that are relevant for human adaptation. To verify this hypothesis, we conducted two studies involving different samples and different measures. We built several models of positive orientation, including additional variables connected to eudaimonia, and checked their fit to the data. In the following paragraphs, we present the results of these studies.

## Method

To measure hedonic aspect of positive orientation, we used three measures proposed by Caprara (2009): *Rosenberg*’*s Self*-*Esteem Scale*, *Satisfaction with Life Scale and Life Orientation Test*-*Revised. The active aspect of meaning of life was measured by the new method*, *called the Life Engagement Test. To measure sense of meaning*, *we used two well*-*known questionnaires*: *Purpose in Life Test* and *Life Attitudes Profile*—*Revised*.

### Measures of Positive Orientation

*Rosenberg*’*s Self*-*Esteem Scale* (*SES*), by Rosenberg (1965), is a simple measure of global self-esteem containing 10 items with a four-point answer scale: *strongly agree* (1) to *strongly disagree* (4). In the current study, internal consistency of the SES is sufficient; the Cronbach’s *α* is between .85 and .86.

*Satisfaction with Life Scale* (*SWLS*), by Diener et al. (1985), is a five-item tool used for assessing life satisfaction on a seven-point Likert response scale from *strongly disagree* (1) to *strongly agree* (7). Reliability of the scale is satisfactory; Cronbach’s *α* is between .75 and .83.

*Life Orientation Test*-*Revised* (*LOT*-*R*), by Scheier et al. (1994), is a 10-item questionnaire used for measuring optimism with a five-point answer scale: six items are diagnostic and four are masking positions. The scale has sufficient internal consistency and Cronbach’s *α* is between .72 and .78.

All three aforementioned scales were translated, validated, and published in Poland (Dzwonkowska et al. 2008; Juczyński 2001).

### Measures of Meaning in Life

*Purpose in Life Test* (*PIL*), by Crumbaugh and Maholick (1981), is a 20-item scale designated to measure meaning of life. Each item is a sentence with opposite endings presented, and a scale from 1 to 7 used to rate them. For example: “My personal existence is: 1—*meaningless*, *without purpose* to 7—*purposeful*, *meaningful*.” The scale has proper reliability; the Cronbach’s *α* = .92.

*Life Engagement Test* (*LET*), by Scheier et al. (2006), is a 10-item scale measuring purpose in life, defined as engagement with personally valuable activities. The scale has satisfactory reliability (Cronbach’s *α* = .72–.87, test-retest reliability *r*_*tt*_ = .61–.76). Validity of the LET was confirmed by correlations to optimism, .39–.61; self-esteem, .41–.61; and life satisfaction, .34–.58 (Scheier et al. 2006).

*Life Attitudes Profile*—*Revised* (*LAP*-*R*), by Reker (1992), is a 48-item questionnaire devoted to measure six existential attitudes, including existential vacuum, life control, death acceptance, goal seeking, purpose, and coherence. LAP-R is not a uniform scale. Exploratory factor analysis usually (as is also the case in our study) derives two higher order factors: the first consists of purpose, coherence, and existential vacuum (reversed) subscales, and the second consists of life control, goal seeking, and death acceptance. The first factor is the basis for a personal meaning index that represents a sense of meaning in life. Since, in the case of this method, we are primarily interested in personal meaning, we included only these three variables in our models, which are loaded on the first factor. Life control, death acceptance, and goal seeking subscales were excluded of our analysis because, apart from meaning of life, they seem to contain a sense of mastery over circumstances and internal locus of control (life control), developmentally conditioned ability to manage with terror of death anxiety (death acceptance) (see e.g., Oleś 1999) and non-acceptance of current routine in life (goal seeking). Cronbach’s *α* = .69–.88 for the Polish version, showing satisfactory internal consistency.

### Participants

The first group consisted of 159 students (95 females), representing a few universities located in Lublin and the south eastern part of Poland. They were aged from 19 to 26 years old (*M* = 21.08; SD = 1.82). They studied various disciplines: Humanities, Social Sciences, Technology, Economics, Biology, or Medicine.

The second group consisted of 200 students (100 females) studying at several universities in different parts of Poland. They were aged from 19 to 31 years old (*M* = 22.77 years). Most of the participants were not married (94.5%), a few were married (5%), and a few were divorced (.5%).

### Procedure

All participants from both groups were asked to answer four or five questionnaires, given in random order, including three measures of positive orientation and one or two measures of the meaning of life. Besides the positive orientation measures, the first sample filled in two instruments, Purpose in Life and Life Attitude Profile—Revised, and the second sample took the Life Engagement Test. Originally, the questionnaires were distributed to 200 students (sample 1) and 248 students (sample 2). Due to participant drop out, as well as incomplete answers, the first sample consisted of 159 participants and the second of 200 participants. The participants were not rewarded for their effort and they were recruited by using the snowball sampling method. Informed consent was obtained from all individual participants included in the study.

### Data Analysis

To verify our hypothesis of whether meaning of life is a significant facet of positive orientation, we specified extended models of positive orientation, checked their fitness to data in each of the samples, and, then, compared them to the basic models of positive orientation (only including self-esteem, satisfaction with life, and optimism). As the compared models are not nested in each other, we used two information indices of goodness of fit, Akaike Information Criterion (AIC), and Bayesian Information Criterion (BIC) that serve to select the best among non-nested models (which usually has lower AIC and BIC; Burnham and Anderson 2002). In sample 1, we separately compared two extended models of positive orientation to the basic model; the first included scores of the PIL and the second included scores of the LAP-R. In sample 2, we compared the basic model with the model that includes the other measure for meaning of life, LET.

Taking into account the limited number of participants in both samples and our main aim of the study (i.e., investigation of relations between latent variables representing different constructs), we decided to aggregate items into parcels before conducting a second order confirmatory factor analysis (Little et al. 2002). In the cases of SES, LOT-R, SWLS, and LET tests, we aggregated items into two parcels per scale. In the case of PIL, which consists of many more items than the others methods, we used three parcels. As LAP-R has been constructed explicitly as a multidimensional questionnaire, we treated each of its scales as an independent indicator of the latent variable, namely the meaning of life. Some studies suggests that SES and LOT-R have a latent structure consisting of two factors referring to how the items are formulated, that is, whether they are positively or negatively worded (Alessandri et al. 2010; Alessandri et al. 2015). To confirm this assumption, we made EFA using our data, and it, indeed, proved the complex structure of SES and LOT-R. Consequently, we aggregated negatively and positively worded items from these methods into separate, homogeneous parcels. However, in the case of LET and PIL, EFA suggested unidimensional latent structures, therefore we aggregated items from each of these questionnaires randomly.

We estimated parameters of models using maximum likelihood procedure. To evaluate model fit, we used the comparative fit index (CFI; Bentler 1990), adjusted goodness-of-fit index (AGFI; Tabachnick and Fidell 2007), and Tucker-Lewis index (TLI; Tucker and Lewis 1973), all indicating acceptable fit if their values exceed .90. We also used the root mean squared error of approximation (RMSEA; Browne and Cudeck 1993) with acceptable values if less than .08. All analyses were made using IBM SPSS AMOS 22 software.

## Results

In Table 1, we present means, standard deviations, and correlations for all variables used in both samples.

**Table 1:** Descriptive statistics and correlations between variables

	Sample 1 (*N* = 159)		Sample 2 (*N* = 200)		Sample 1 R Pearson’s correlations			Sample 2 R Pearson’s correlations		
	Mean	SD	Mean	SD	SES	LOT-R	SWLS	SES	LOT-R	SWLS
SES	30.3	4.5	23.7	4.8	–			–		
LOT-R	15.2	4.7	14.6	4.5	.55	–		.65	–	
SWLS	20.9	5.7	21.2	5.1	.50	.48	–	.56	.55	–
LET	–	–	23.8	3.9	–	–	–	.53	.49	.48
PIL	104.7	17.8	–	–	.68	.59	.62	–	–	–
PU	40.7	8.1	–	–	.52	.53	.51	–	–	–
CO	39.9	8.1	–	–	.45	.52	.49	–	–	–
EV	28.5	8.2	–	–	−.51	−.41	−.43	–	–	–

All correlations are significant on *p* < .001

*SES* Self-Esteem Scale, *LOT*-*R* Life Orientation Test, *SWLS* Satisfaction With Life Scale, *LET* Life Engagement Scale, *PIL* Purpose In Life, *PU* Purpose Scale, *CO* Coherence Scale, *EV* Existential Vacuum Scale

First, we present two results obtained in the first group (*N* = 159). The baseline model, including self-esteem, satisfaction with life, and optimism, fitts very well to the data (see Table 2). All loadings are high and comparable between variables, replicating results from other studies (e.g., Oleś et al. 2013), and supporting the idea of positive orientation as a latent factor that explains individual differences in the tendency to positively evaluate the self, life, and future.

**Table 2:** Extended models of positive orientation

	Chi2(*df*)	*p*=	AIC	BIC	CFI	TLI	RMSEA (95% CI)
Sample 1							
Baseline	9.34 (6)	.16	39.3	85.4	.99	.98	.06 (.001–.13)
Model 1	26.19 (23)	.29	70.2	137.7	.99	.99	.03 (.001–.07)
Model 2	26.18 (22)	.24	72.2	142.8	.99	.99	.04 (.001–.08)
Sample 2							
Baseline	7.66 (6)	.26	37.7	87.1	.99	.99	.04 (.001–.10)
Model 3	17.43 (16)	.36	57.4	123.4	.99	.99	.02 (.001–.07)

*Baseline model* PO + SES + LOT-R, *Model 1* PO + SES + LOT-R + PIL, *Model 2* PO + SES + LOT-R + LAP-R (PU + CO + EV), *Model 3* PO + SES + LOT-R + LET

**Fig. 1:**
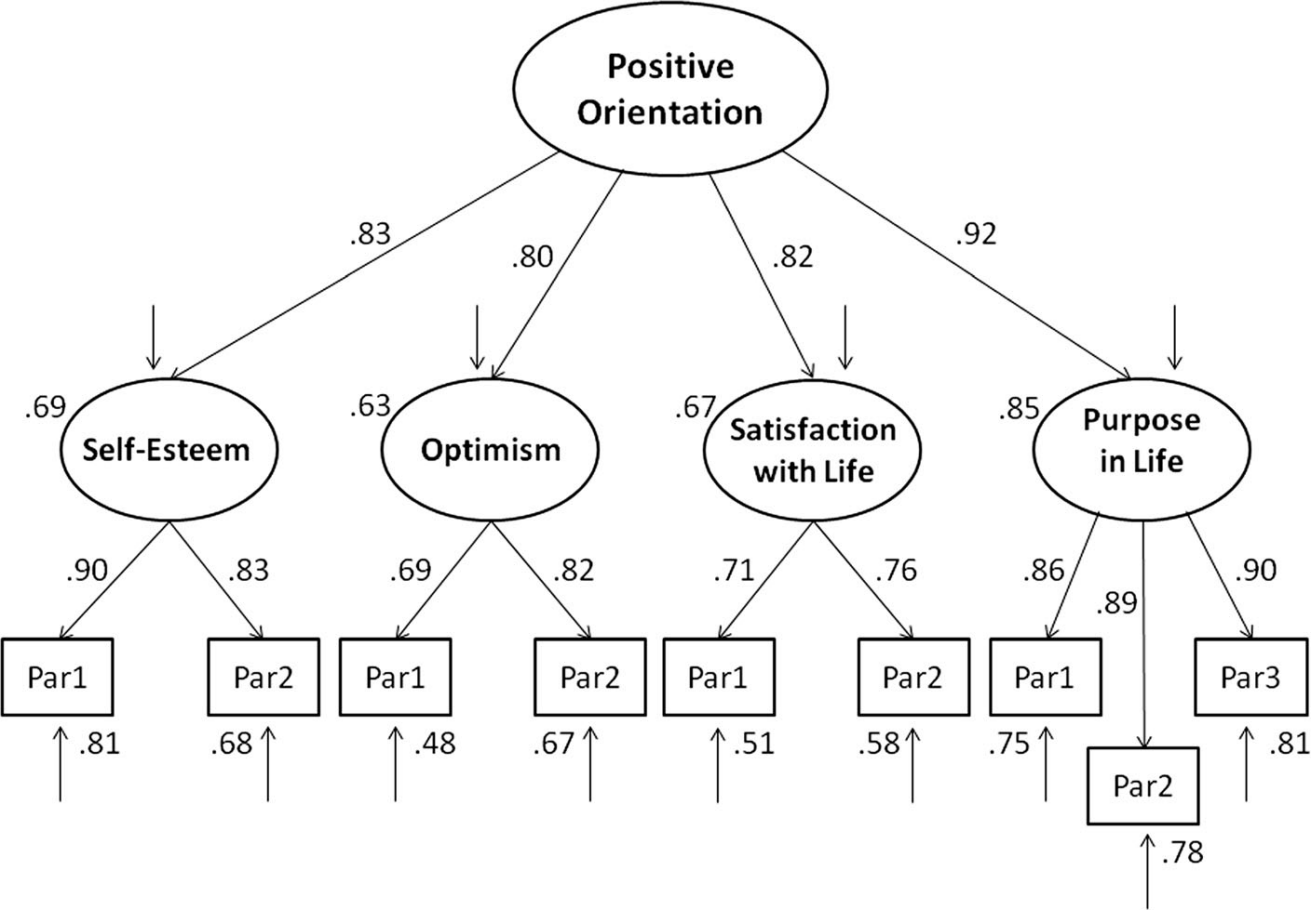
Structural model of positive orientation including self-esteem (SES), life satisfaction (SWLS), optimism (LOT), and purpose in life (PIL)

The model of positive orientation, extended to meaning of life as measured by the PIL, also fit very well to the data (see Fig. 1). However, comparison of AIC and BIC indices indicated that the baseline model is better than the extended model (see Table 2).

**Fig. 2:**
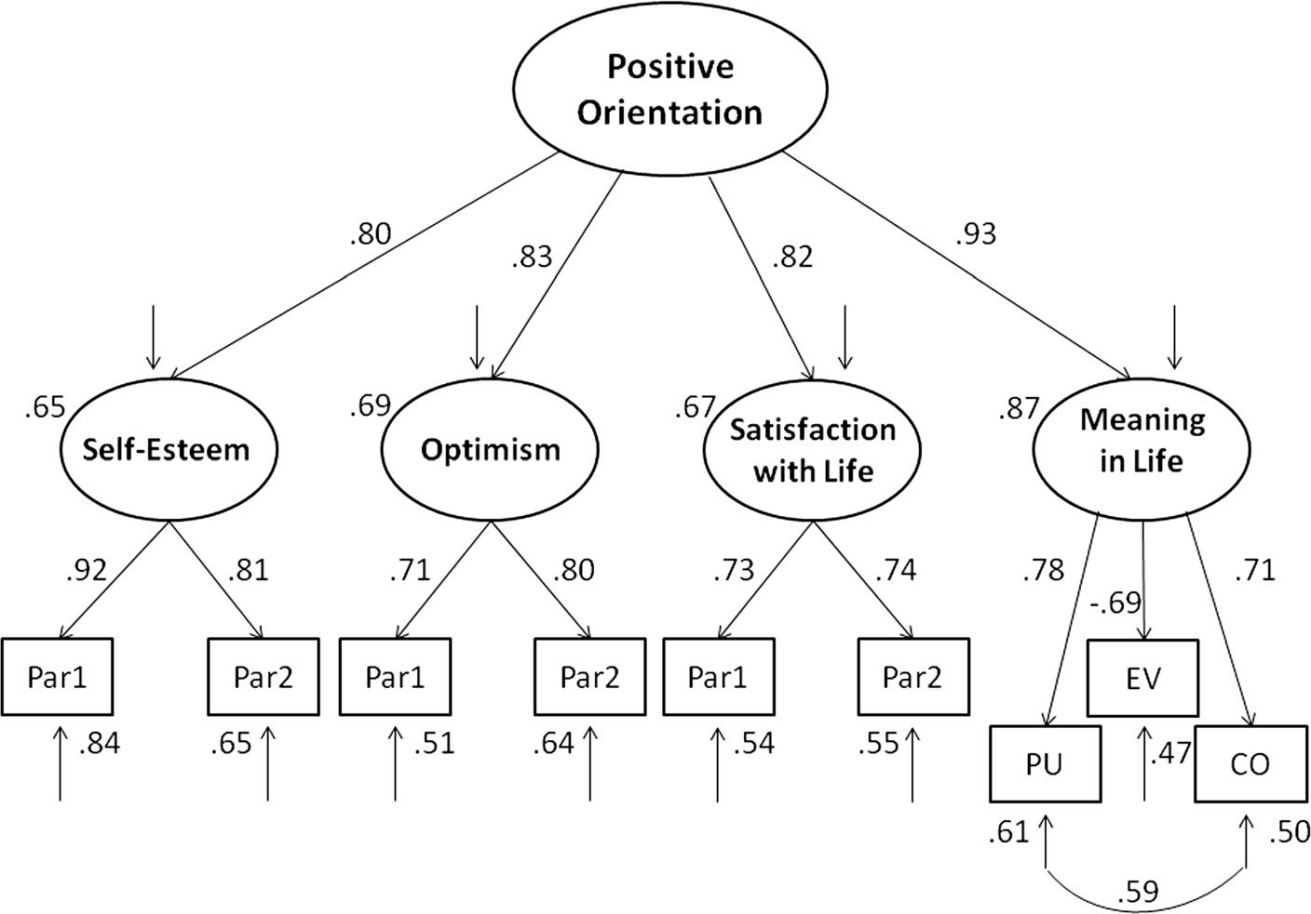
Structural model of positive orientation including self-esteem (SES), life satisfaction (SWLS), optimism (LOT), and shortened life attitudes profile—revised (LAP-R)

In the same sample, another extended model was verified. This model implies that positive orientation consists of four variables: self-esteem, satisfaction with life, optimism, and meaning of life; however, this time the last variable was measured by Life Attitudes Profile—Revised. Three LAP-R scales, Purpose, Coherence, and Existential Vacuum, were treated as separate indicators of the latent variable named meaning of life (these scales were chosen because they refer to the Personal Meaning Index formulated by Reker (1992) and share high common variance). The specified model fits very well to the data. However, comparison of AIC and BIC indices indicated, again, that the baseline model is better than the extended model (see Table 2 and Fig. 2).

**Fig. 3:**
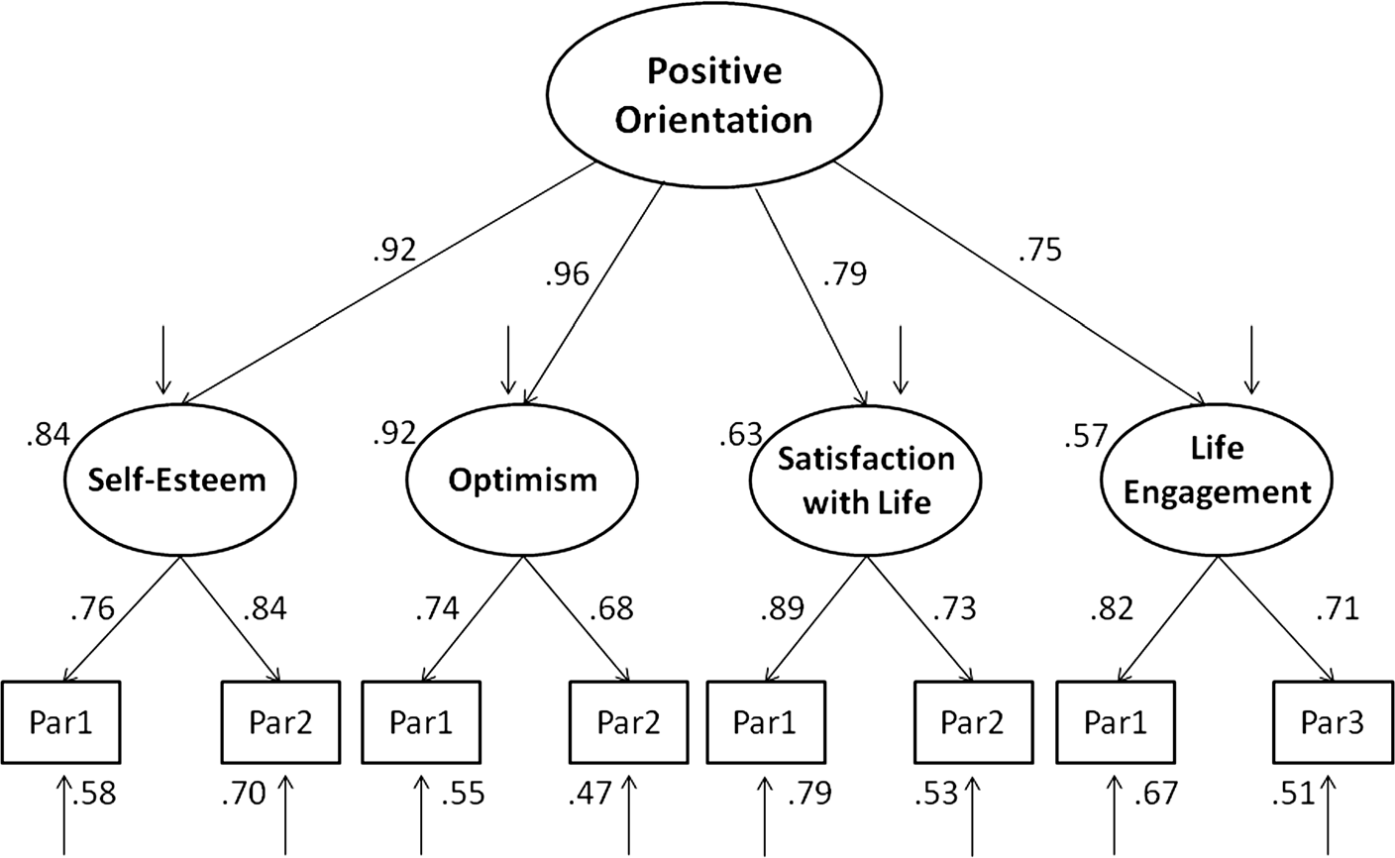
Structural model of positive orientation including self-esteem (SES), life satisfaction (SWLS), optimism (LOT), and life engagement (LET)

For the second sample (*N* = 200), the third model was verified, this time including engagement in life (LET), which actually also refers to purpose in life. As Scheier et al. (2006) argue, “The LET was specifically designed to assess purpose in life by assessing extents to which people engaged in activities that they found valuable and significant. We focused the LET in this way because we believe it is this aspect of purpose in life that is critical to defining the construct” (pp. 294–295). Compared to PIL and LAP-R, the Life Engagement Scale measures more active aspects of meaning of life, i.e., how it manifests in human goal-directed activity. As in the case of previous models, we specified this model with four latent variables representing self-esteem, satisfaction with life, optimism, and engagement in life. Then, we tested its accuracy with goodness-of-fit and factor loadings. This model also fits the data very well (see Table 2 and Fig. 3). Compared to the baseline model, as it was in the case of previous models, it proved to be a worse fit (see Table 2).

Summing up, all three models involve not only self-esteem, satisfaction with life, and optimism but also meaning of life, as measured by three different tools, which fitted to the data pretty well. However, informational criteria of fitness (AIC and BIC) indicated the baseline model as preferred, for each case. We will discuss these results in the next section.

## Discussion

Positive orientation is the name given to what life satisfaction, self-esteem, and optimism have in common. It is a stable mode of facing reality, of reflecting upon and processing experiences, and of framing events (Caprara et al. 2009). A core feature of the notion of positive orientation is that viewing oneself, one’s life, and one’s future optimistically predisposes people to master life challenges, despite obstacles, failures, and losses.

The extended model of positive orientation, constituted from self-esteem, life satisfaction, optimism, and purpose in life, fits the data very well. This means there is a common basis for all four variables, which points to a general inclination to evaluate the self, life, future, and purpose in life in a positive way. It also suggests that positive orientation, as composed of only three facets, does not represent a full range of variables underlying positive functioning. It is probable that positive orientation not only involves an inclination to positively evaluate the self and some important aspects of life and future, it also overwhelms personally appreciated goals and activities, implying that life is worth living. All our analyses show the same results.

Most of the time, when two models fit the data well, the more parsimonious model is preferred over the more complex one (Vandekerckhove et al. 2015). In this case, we argue something opposite. Note, that the aim of this study was not to find the simplest model of positive orientation but to check if positive orientation, as latent variable, can explain more than three variables, not only self-esteem, satisfaction with life, and optimism, but also meaning of life. The preference of the baseline models consisting of only three variables is understood from a statistical point of view because such models have fewer free parameters than extended ones. This means that the baseline models are more parsimonious; however, on the other hand, they provide no information about common variance shared by hedonic and eudaimoinc aspects of well-being. Therefore, taking into account that the extended models fit data well, we claim that they are a better explanation of the essence of positive orientation.

Meaning of life, assessed by different measures (PIL, LAP-R, LET) and measured in different groups, constitutes a latent variable, together with the triad responsible for positive orientation in previous studies (Caprara 2009). Theoretically, such findings are fully justified. First, all these variables relate to positive aspects of thinking. Second, they all represent important human needs, such as having high self-esteem, living a good and valuable life, having a stable and foreseeable future, and being able to find purpose and meaning in life (see e.g., Epstein 1990). Third, they all refer to the self, that is, they come from evaluation of *my* person, *my* life, *my* activity, and *my* future. Fourth, they all represent important and complementary aspects of healthy functioning and adaptation (Heintzelman and King 2014; Ryff 2014). In sum, we argue that common basis of self-esteem, optimism, satisfaction with life, and meaning in life—positivity of self and life—underlines adaptive functioning. Moreover they constitute sufficient conditions of happiness, as it is understood both in hedonic and eudemonic senses; thus, they play a crucial role in human adaptation.

Posing the question as to why these four variables seems crucial, we propose to extend the discussion on a core meaning of positive orientation referring to both sociobiological (Heine et al. 2006) and existential (Todres and Galvin 2010) concepts of human beings. On the one hand, positive inclination of thinking promotes expansive adaptation, seeking new activities, experiences, and life circumstances. On the other hand, three or four (or maybe more) facets of positive orientation suggest the presence of a general tendency to similarly evaluate important meanings of the self and the external world, as well as their relationships. Heine et al. (2006) proposed the meaning maintenance model as a hypothetical explanation for coherence of social motivation, and their model can explain coherence of beliefs and evaluations of the self and the external world. The person as meaning-maker composes units of internal and external reality into one system, using meanings conceived as relationships. Meanings as relationships serve as a common basis for beliefs concerning the self, life, and future. Moreover, Heine et al. (2006) propose fluid compensation among four domains of meaning making and basic needs at the same time―self-esteem, certainty, belongingness, and symbolic immortality. Aforementioned domains are not strictly connected but they function as a more or less coherent system, which can explain affinity of assessments concerning the self, life satisfaction, purposefulness, and the future. Note that positive concepts of the future, self, or life overcome uncertainty; similar rules apply to belongingness, which implies well-being, self-esteem, and meaningful life.

Another existential notion explains positive orientation as a phenomenon belonging to awareness, namely “dwelling-mobility” existential theory by Todres and Galvin (2010). According to this theory, well-being emerges from two opposed sources: excitation preceding or following purposeful activity (mobility aspect), and acceptance or even contemplation of “here and now” (dwelling aspect). “The deepest possibility of existential well-being lies in the unity of dwelling-mobility” (Todres and Galvin 2010, p. 5). This is exactly what we found: the combination of self-esteem, life satisfaction (derived from active life or passive enjoyment), optimism, and life engagement constitute positive orientation that, in this context, can be interpreted as a dispositional basis of the unity of “dwelling-mobility.”

Providing that positive orientation underlines personal inclination for positive evaluation of one’s life, self, and future, we postulate that, besides life conditions, events, or realization of chosen strivings, there is a natural basis for both hedonistic and eudemonic senses of happiness.

The studies presented in this article have some limitations. First of all, they are based on limited samples of graduate students, which makes generalization of results problematic. As Frankl claimed (1963) and Reker and others showed (1987), older people’s personal meaning system is more integrated and consolidated than younger people. In consequence, older people have a tendency to experience greater meaning in life (Reker and Fry 2003). How developmental processes can modify significance of meaning in life in the context of positive orientation should be a subject of further research using different (older) samples. We can only speculate that, in older people, the essence of positive orientation may evaluate toward meaning of life to a greater extent than in younger people.

Investigation of conditions in which assembled “symmetry” of self-esteem, optimism, satisfaction with life, and meaning of life might be “broken” seems particularly interesting. This would mean that all of the variables might have separate, unrelated functions and, therefore, do not create a common latent factor of positive orientation. It is probable that such a situation might be observed in clinical samples.

## References

[CR1] Alessandri G, Vecchione M, Fagnani C, Bentler P, Barbaranelli C, Medda E, Nisticò L, Stazi MA, Caprara GV (2010). Much more than model fitting? Evidence for the heritability of method effect associated with positively worded items of the Life Orientation test Revised. Struct Equ Model.

[CR2] Alessandri G, Vecchione M, Eisenberg N, Łaguna M (2015). On the factor structure of the Rosenberg (1965) general self-esteem scale. Psychol Assess.

[CR3] Bandura A (2001). Social cognitive theory: an agentic perspective. Annu Rev Psychol.

[CR4] Bentler PM (1990). Comparative fit indexes in structural models. Psychol Bull.

[CR5] Browne MW, Cudeck R, Bollen KA, Long JS (1993). Alternative ways of assessing model fit. Testing structural equation models.

[CR6] Burnham KP, Anderson DR (2002). Model selection and multimodel inference.

[CR7] Caprara GV (2009). Positive orientation: turning potentials into optimal functioning. Bull Eur Health Psychol.

[CR8] Caprara GV, Fagnani C, Alessandri G, Steca P, Gigantesco A, Sforza LLC, Stazi MA (2009). Human optimal functioning: the genetics of positive orientation towards self, life and the future. Behav Genet.

[CR9] Caprara GV, Steca P, Alessandri G, Abela JRZ, McWhinnie CM (2010). Positive orientation: explorations on what is common to life satisfaction, self-esteem, and optimism. Epidemiologia e Psichiatria Sociale.

[CR10] Crumbaugh JC, Maholick LT (1981). Manual and instructions for the Purpose-in-Life Test.

[CR11] Deci EL, Ryan RM (2008). Hedonia, eudaimonia, and well-being: an introduction. J Happiness Stud.

[CR12] Delle Fave A, Brdar I, Freire T, Vella-Brodick D, Wissing MP (2011). The eudaimonic and hedonic components of happiness: qualitative and quantitative findings. Soc Indicators Res.

[CR13] Diener E, Emmons RA, Larsen RJ, Griffin S (1985). The satisfaction with life scale. J Pers Assess.

[CR14] Dzwonkowska I, Lachowicz-Tabaczek K, Łaguna M (2008). *Samoocena i jej pomiar. Skala samooceny SES M. Rosenberga* [Self-esteem and its measurement. Self-Esteem Scale by M. Rosenberg].

[CR15] Epstein S, Pervin L (1990). Cognitive-experiential self-theory. Handbook of personality theory and research: theory and research.

[CR16] Feder A, Southwick SM, Goetz RR, Wang Y, Alonso A, Smith BW, Buchholz KR, Waldeck T, Ameli R, Moore J, Hain R, Charney DS, Vythilingam M (2008). Psych: Int Biol Proc.

[CR17] Frankl VE (1963). Man’s search for meaning.

[CR18] Frankl VE (1984). The unheard cry for meaning: psychotherapy and humanism.

[CR19] Fredrikson BL, Losada MR (2005). Positive affect and the complex dynamics of human flourishing. Am Psychol.

[CR20] Heine SJ, Proulx T, Vohs KD (2006). Meaning maintenance model: on the coherence of human motivations. Personal Soc Psychol Rev.

[CR21] Heintzelman SJ, King LA (2014). Life is pretty meaningful. Am Psychol.

[CR22] Heisel MJ, Flett GL (2004). Purpose in life, satisfaction with life and suicide ideation in a clinical sample. J Psychopathol Behav Assess.

[CR23] Ho MY, Cheung FM, Cheung SF (2010). The role of meaning in life and optimism in promoting well-being. Personal Individ Differ.

[CR24] Jacobsen B (2007). Invitation to existential psychology. A psychology for the unique human being and its applications in therapy.

[CR25] Juczyński Z (2001). *Narzędzia pomiaru w promocji i psychologii zdrowia* [Measurement tools in promotion and psychology of health].

[CR26] Judge TA, Erez A, Bono JE, Thoresen CJ (2003). The core self-evaluations scale: development of a measure. Pers Psychol.

[CR27] Kahneman D, Diener E, Schwarz N (1999). Well-being: the foundations of hedonic psychology.

[CR28] Land KC, Michalos AC, Sirgy MJ (2012). Handbook of social indicators and quality of life research.

[CR29] Little TD, Cunningham WA, Shahar G, Widaman KF (2002). To parcel or not to parcel: exploring the question and weighing the merits. Struct Equ Model.

[CR30] Lyubomirsky S, Tkach C, DiMatteo MR (2006). What are the differences between happiness and self-esteem?. Soc Indic Res.

[CR31] Oleś PK (1999). Towards a psychological model of midlife crisis. Psychol Rep.

[CR32] Oleś M (2014). Factor structure of quality of life in adolescents. Psychol Rep.

[CR33] Oleś PK, Alessandri G, Oleś M, Bąk W, Jankowski T, Laguna M, Caprara GV (2013). Positive orientation and generalized self-efficacy. Stud Psychol.

[CR34] Peterson C, Park N, Seligman ME (2005). Orientations to happiness and life satisfaction: the full life versus the empty life. J Happiness Stud.

[CR35] Pinquart M, Frohlich C (2009). Psychosocial resources and subjective well-being of cancer patients. Psychol Health.

[CR36] Reker GT (1992). Manual of the Life Attitude Profile-Revised (LAP-R).

[CR37] Reker GT, Fry PS (2003). Factor structure and invariance of personal meaning measures in cohort of younger and older adults. Personal Individ Differ.

[CR38] Reker GT, Peacock EJ, Wong PTP (1987). Meaning and purpose in life and well- being: a life-span perspective. J Gerontol.

[CR39] Rosenberg M (1965). Society and the adolescent image.

[CR40] Ryan RM, Huta V, Deci EL (2008). Living well: a self-determination theory perspective on eudaimonia. J Happiness Stud.

[CR41] Ryff C (2014). Psychological well-being revisited: advances in the science and practice of eudaimonia. Psychother Psychosom.

[CR42] Scheier MF, Carver CS, Bridges MW (1994). Distinguishing optimism from neuroticism (and trait anxiety, self-mastery, and self-esteem): a reevaluation of the Life Orientation Test. J Pers Soc Psychol.

[CR43] Scheier MF, Wrosch C, Baum A, Cohen S, Martire LM, Matthews KA, Schulz R, Zdaniuk B (2006). The life engagement test: assessing purpose in life. J Behav Med.

[CR44] Schlegel RJ, Hicks JA, Arndt J, King LA (2009). Thine own self: true self-concept accessibility and meaning in life. J Pers Soc Psychol.

[CR45] Schueller SM, Seligman MEP (2010). Pursuit of pleasure, engagement, and meaning: relationships to subjective and objective measures of well-being. J Positive Psychol.

[CR46] Seligman MEP (2002). Authentic happiness: using the new positive psychology to realize your potential for lasting fulfillments.

[CR47] Steger MF, Frazier P (2005). Meaning in life: one link in the chain from religion to well-being. J Couns Psychol.

[CR48] Steger MF, Kashdan TB (2007). Stability and specificity of meaning in life and life satisfaction over one year. J Happiness Stud.

[CR49] Tabachnick BG, Fidell LS (2007). Using multivariate statistics.

[CR50] Taylor SE, Brown JD (1988). Illusion and well-being: a social psychological perspective on mental health. Psychol Bull.

[CR51] Todres, L., & Galvin, K. (2010). “Dwelling-mobility”: an existential theory of well-being. *International Journal of Qualitative Studies on Health and Well-Being, 5*(3). doi:10.3402/qhw.v5i3.5444. 10.3402/qhw.v5i3.5444 PMC2938924 20842215

[CR52] Tucker L, Lewis C (1973). A reliability coefficient for maximum likelihood factor analysis. Psychometrika.

[CR53] Vandekerckhove J, Matzke D, Wagenmakers EJ, Busemeyer JR, Townsend JT, Wang ZJ, Eidels A (2015). Model comparison and the principle of parsimony. Oxford handbook of computational and mathematical psychology.

[CR54] Vaughan SM, Kinnier RT (1996). Psychological effects of a Life Review intervention for persons with HIV disease. J Couns Dev.

[CR55] Vella-Brodric DA, Park N, Peterson C (2009). Three ways to be happy: pleasure, engagement, and meaning—findings from Australian and US samples. Soc Indic Res.

